# Ciprofloxacin-Induced Reaction Imitating a Lupus Flare: A Case Report

**DOI:** 10.7759/cureus.8327

**Published:** 2020-05-28

**Authors:** Adnan Liaqat, Aisha Barlas, Talal Barlas, Hamna Khurram, Hamza Liaqat

**Affiliations:** 1 Internal Medicine, Southeast Health Medical Center, Alabama, USA; 2 Internal Medicine, Mercy Health, Rockford, USA; 3 Internal Medicine, The Wright Center, Scranton, USA; 4 Internal Medicine, Wah Medical College, Wah Cantonment, PAK

**Keywords:** ciprofloxacin skin reaction, lupus flare, ciprofloxacin, systemic lupus erythematosus

## Abstract

Systemic lupus erythematosus (SLE) is an autoimmune disease that can affect almost any organ in the body. It usually runs a chronic course with systemic inflammation, and age at diagnosis varies from 15 to 44 years. Laboratory reports often show high anti-nuclear antibody (ANA) levels, increased anti-double-stranded deoxyribonucleic acid (anti-dsDNA) levels, and low complement levels. 'Lupus flare' is a term used for an acute exacerbation of previously existing SLE. It usually manifests as an acute worsening of clinical signs and symptoms, along with an abrupt change in typical laboratory values. Triggers for a lupus flare include viral or bacterial infections, acute stress, and various environmental factors such as ultraviolet (UV) light. Ciprofloxacin is a broad-spectrum fluoroquinolone antibiotic used for various bacterial infections. On rare occasions, ciprofloxacin can cause adverse effects in the body, which may resemble an acute flare of SLE symptoms in patients with previously controlled disease. We have presented such a case of ciprofloxacin-induced reactions mimicking a lupus flare in an SLE patient.

## Introduction

Systemic lupus erythematosus (SLE) is a chronic multi-systemic disease of autoimmune origin. It has a relapsing-remitting course, and its disease pattern, ranging from mild to severe, has an association with high morbidity and mortality. A lupus flare is an acute worsening of signs/symptoms and laboratory parameters in an SLE patient. Symptoms can be unpredictable, and it can affect multiple organs, resulting in a need to alter the treatment strategy to achieve control of disease progression. Although some patients experience flares during a disease course that often result in poor outcomes, the overall rate of survival has increased in recent years because of advancements in diagnostic methods, treatment strategies, and early identification of complications [1-2]. Lupus flares can occur during the disease course, and the management strategy should revolve around avoiding risk factors along with early diagnosis and treatment [3]. Emotional stress, non-compliance with drug treatment, infections, surgery, pregnancy, and exposure to sunlight are a few risk factors for triggering a lupus flare. There is no accurate diagnostic test available for diagnosing lupus flares, but anti-double-stranded deoxyribonucleic acid (anti-ds DNA) levels show disease activity along with complement levels. Clinical judgment is usually a way to diagnose exacerbations. Some presentations can include worsening of skin findings, increased fatigue, arthralgias, severe headache and abdominal pain, a sudden drop in hemoglobin, arrhythmias, new-onset hematuria, or acute psychosis [4-5].

Central nervous system (CNS) involvement can also present with seizures or chorea. In pregnancy, lupus flare can cause miscarriages, especially in the presence of serum antiphospholipid antibodies [6]. A few cases have reported ciprofloxacin as a cause of adverse reactions, with symptoms ranging from gastrointestinal (GI) disturbances, seizures, and the onset of a recent rash [7-10]. Similar reports have previously shown allergic reactions immediately after the first dose [11]. Rarely (0.1% only), it can present with arthralgias and myalgias [7-10]. In our case, an SLE positive patient presented with a urinary tract infection, and we prescribed a course of ciprofloxacin. On the third day, the patient presented with symptoms that resembled a lupus flare but were possibly because of ciprofloxacin's adverse reaction.

## Case presentation

Our case is that of a 34-year-old Southeast Asian female with a two-year history of SLE, which initially manifested with arthralgias, malar rash, anemia, and proteinuria, and she was diagnosed with positive anti-nuclear antibodies, low complement levels, and increased anti-ds DNA levels. She had good control over her disease with low-dose prednisolone and hydroxychloroquine. During her two-year disease course, she suffered from upper respiratory tract infections and urinary infections multiple times, along with intermittent arthralgias. During this visit, she presented in the outdoor patient department with a complaint of low-grade fever and burning micturition for the previous two days.

On a general physical examination, the patient looked oriented to time, place, and person. Her temperature was 101°F, pulse 90/min, and BP 125/80 mmHg. Examination of her oral cavity revealed a few aphthous ulcers, and the classic butterfly rash of SLE was evident on her face. There were no significant findings during the systematic examination.

Laboratory investigations revealed Hb 9.9 g/dl with mean corpuscular volume (MCV) 70 fL, white blood cell (WBC) 16 × 103 cells/UL (75% neutrophils, 20% lymphocytes, 3% monocytes, 1% eosinophils), and erythrocyte sedimentation rate (ESR) was 20 mm/hr. C-reactive protein (CRP) was 5 mg/dl. Her urinalysis showed >10 WBC/high power field (HPF), positive nitrites, and urinary pH 5.5. No proteinuria or red blood cells (RBCs) were observed on the urine exam. The blood urea nitrogen (BUN) was 22 mg/dl, and serum creatinine was 0.9 mg/dl. There was no evidence of lupus nephritis. We also took blood and urine samples for culture and sensitivity. Urinary tract infection was the diagnosis, and we prescribed ciprofloxacin 500 mg PO q12hr along with acetaminophen for fever. We also counseled the patient about maintaining adequate hydration.

After 48 hours of starting ciprofloxacin, the patient showed up in the emergency department with her family with the complaint of severe headache, generalized body aches, and pain in both knees and shoulder joints. On examination, we observed a prominent maculopapular rash on her chest and back. On admission, her BP was 130/90 mmHg, pulse was 92/min, temperature was 98.9°F, and RR was 18/min. Laboratory investigation showed hemoglobin was 9.7 g/dl, WBC count was 13.7 × 103 cells/UL (61% neutrophils, 30% lymphocytes, 6% monocytes, 2% eosinophils). ESR and CRP were 18 mm/hr and 4 mg/dl, respectively. A repeat urinalysis revealed <6 WBC/HPF, negative nitrites, urine PH 5.2, and traces of protein. The patient's BUN and serum creatinine levels were still within normal limits. The chest radiograph and electrocardiogram (EKG) were normal.

The differential diagnoses considered were ciprofloxacin reaction and lupus flare. Ciprofloxacin seemed likely, keeping in mind the acute onset of maculopapular rash, arthralgias, and headache. We stopped her ciprofloxacin immediately and administered intravenous corticosteroids. Urine culture results from her previous visit showed E. coli sensitive to cefixime and ciprofloxacin. After resolution of the acute symptoms, we discharged her on cefixime and referred her to a rheumatologist for lupus follow-up.

## Discussion

Ciprofloxacin is a broad-spectrum antibiotic that belongs to the second-generation fluoroquinolones and is active against gram-positive and gram-negative bacteria. It is a DNA gyrase and topoisomerase II inhibitor, which results in the inhibition of cell division [12]. Known adverse effects associated with ciprofloxacin use include insomnia, nausea, lightheadedness, headache, dizziness, tendon rupture, and diarrhea [7]. A lupus flare-up directly because of ciprofloxacin use is very rare. However, there were a few cases reported of ciprofloxacin-induced skin reactions, which can look similar to a lupus flare. One case reported toxic epidermal necrolysis in an SLE patient after the use of ciprofloxacin. Upon reviewing several case reports, we found only a few cases that had similar presentations and outcomes to our case report, and we mention those cases in Table 1.

**Table 1:** Literature review of the reported cases of ciprofloxacin-induced reactions in SLE patients SLE: systemic lupus erythematosus; IV: intravenous

Case	Authors	Age/Sex	SLE diagnosis	Time of onset of symptoms	Presentation	Diagnosis	Management	Outcomes
Case 1	Moshfeghi M, et al. [13]	31 y/F	Known case of SLE, didn’t mention the duration of disease	Following her 1st dose	Painful, worsening rash progressed to all over the body leading to desquamation, positive Nikolsky's sign	Toxic epidermal necrolysis	Treated in the burns unit	Recovered after prolonged hospitalization
Case 2	Mysler E, et al. [14]	34 y/F	Diagnosed 15 years ago	Within 2 hours of the first dose	Initially pruritic and a sense of bronchospasm followed by myalgias, arthralgias, and arthritis	Ciprofloxacin reaction mimicking Lupus flare	I/M Epinephrine and Methylprednisolone	Resolved over six days
Case 3	Mysler E, et al. [14]	26 y/F	Diagnosed 16 years ago	Within 48 hours and became worse over the next 24 hours	Generalized arthralgias in the upper and lower limbs	Ciprofloxacin reaction mimicking Lupus flare	Discontinuation of Ciprofloxacin	Resolved over the next several days
Case 4	Mysler E, et al. [14}	36 y/F	Diagnosed 11 years ago	Within 24 hours of initial dose	Upper and lower extremity arthralgias and arthritis	Ciprofloxacin reaction mimicking Lupus flare	30 mg IV Prednisolone, and Ciprofloxacin discontinued	Symptoms resolved in 12 hours
Case 5	Andrew J Newman, et al. [15]	46 y/M	Diagnosed with Subcutaneous SLE after the appearance of this rash	10-12 days after initial dose	Erythematous crescent-shaped plaques over sun-exposed areas	Drug-induced subacute cutaneous lupus erythematosus	Medium potency topical steroids	Resolved at four weeks follow-up

Proposed mechanisms of ciprofloxacin reactions include a difference in the genetics of the cytochrome p-450 system, which may result in toxic metabolites of ciprofloxacin reacting against the immune system. This ciprofloxacin reaction can manifest as fever, myalgias, sudden maculopapular rash, increased prominence of a butterfly rash, arthralgias, or acute onset of hematuria. Laboratory investigations will show raised anti-ds DNA levels. Complement levels can be low to normal and typically resolve in a few days after the discontinuation of ciprofloxacin. The immediate solution to this problem is to stop the offending drug and administer a short course of intravenous corticosteroids if symptoms are severe. Follow-up of such a patient includes anti-ds DNA and complement levels to evaluate the progression of the condition.

We initially considered urinary tract infection (UTI) as the cause of lupus flare symptoms in our patient, as acute infections are a known cause of SLE exacerbations, but the timing of onset of this infection and antibiotic use disproved our notion. The patient had been having UTI symptoms for two days before ciprofloxacin use and showed no signs resembling an SLE flare at that time. The development of her flare-like symptoms (appearance of a maculopapular rash, myalgia, arthralgia) only began in the 48 hours after starting ciprofloxacin.

We suspected ciprofloxacin was causing a reaction mimicking lupus flares [14]. Therefore, we suggest this be kept in mind while monitoring SLE disease activity with anti-ds DNA and complement levels to diagnose lupus flares [16]. If there is a suspicion of ciprofloxacin as the cause of flare-like symptoms, immediately stop the drug and administer corticosteroids to control the excessive activation of the immune system and to induce remission. We feel our case is a significant addition to existing data on this subject.

## Conclusions

Several types of skin reactions with ciprofloxacin have been reported previously in non-SLE patients. This case highlights a rare adverse reaction of ciprofloxacin in SLE patients, which may resemble a lupus flare. The time of symptom onset after using ciprofloxacin can vary from hours to days, and steroids have proven effective as a treatment after stopping ciprofloxacin. Treating physicians need to be careful in differentiating these rare reactions of ciprofloxacin from genuine lupus flares in SLE patients, as their presenting signs and symptoms are similar. We require further studies to prove if ciprofloxacin can be a direct cause of a lupus flare.
